# Supplementary material for: Surface chemistry governs cellular tropism of nanoparticles in the brain
Source: Nat Commun. 2017 May 19;8:15322. doi: 10.1038/ncomms15322 (PMC5454541; doi:10.1038/ncomms15322)
Supplement: Supplementary Information — Supplementary Figures and Supplementary Tables [file ncomms15322-s1.pdf]

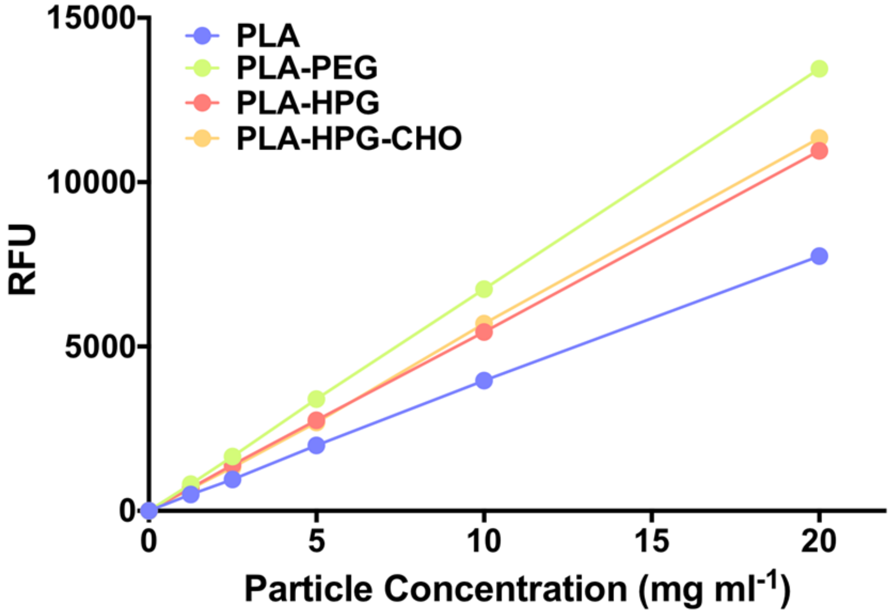

Supplementary Figure 1: DiA dye loading in different NP formulations. Standard curve of particles at different concentrations measuring fluorescence allowed for normalization of FACS data (results are the mean of N = 2 technical replicates with particle loading repeated for each batch of particles).

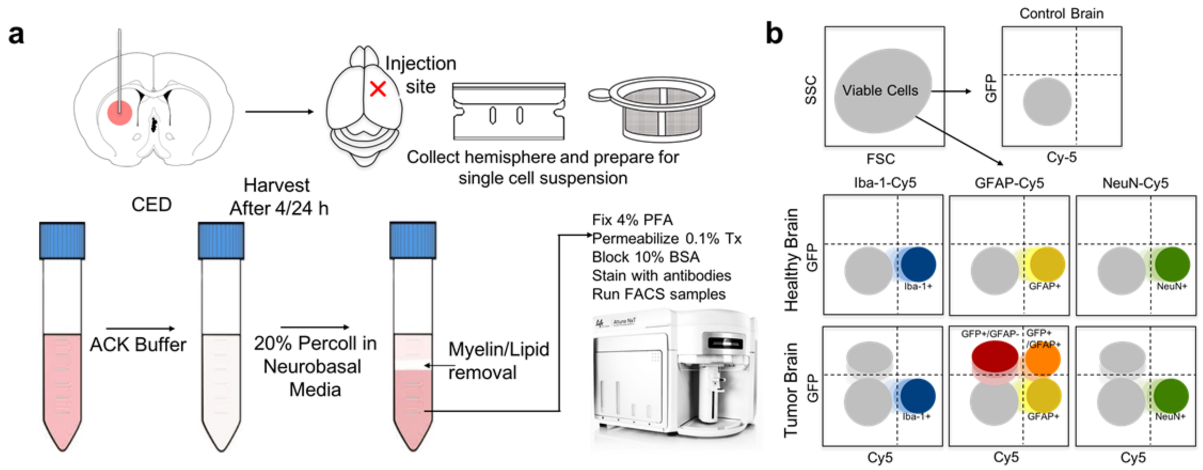

Supplementary Figure 2: Experimental procedure and data analysis. (a) Schematic of sample preparation for flow cytometry. Brain was harvested post CED and processed into single cell suspension. Red blood cells were lysed using ACK lysing buffer and lipid/myelin was removed using a 20% percoll solution. Samples were fixed, permeabilized, blocked and stained for intracellular markers. (**b)** Representative schematic of FACS plot of in vivo sample preparation. Iba-1, GFAP and NeuN conjugated primary antibodies were used to identify microglia, astrocyte and neuron populations respectively for both healthy and tumor bearing rats.

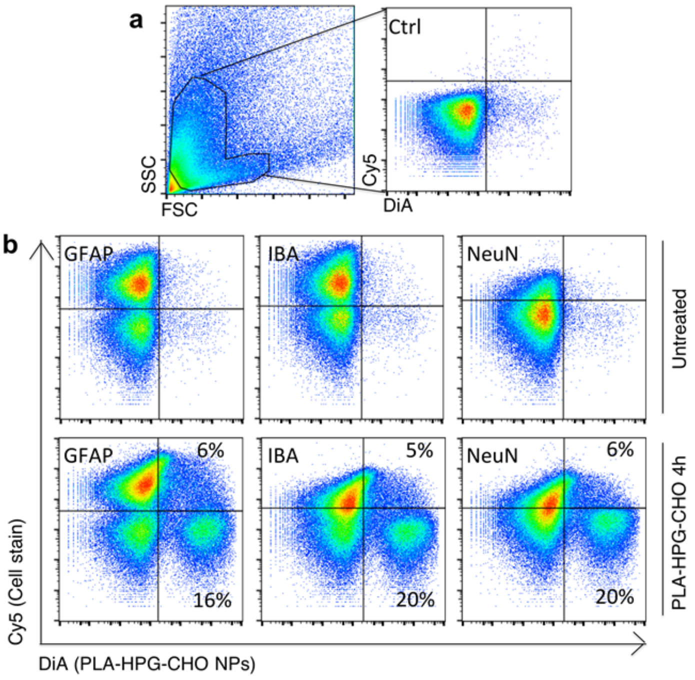

Supplementary Figure 3: Example of dot plots on single planes obtained through data analysis. (a) The cell population is first gated from all the event collected, and plotted using the Cy-5 (y-axis) and DiA channels (x-axis). **(b)** Representative results obtained for each staining in untreated brains (top row) and brains treated for 4 h with PLA-HPG-CHO NPs, showing how the different percentages of cells are extracted.

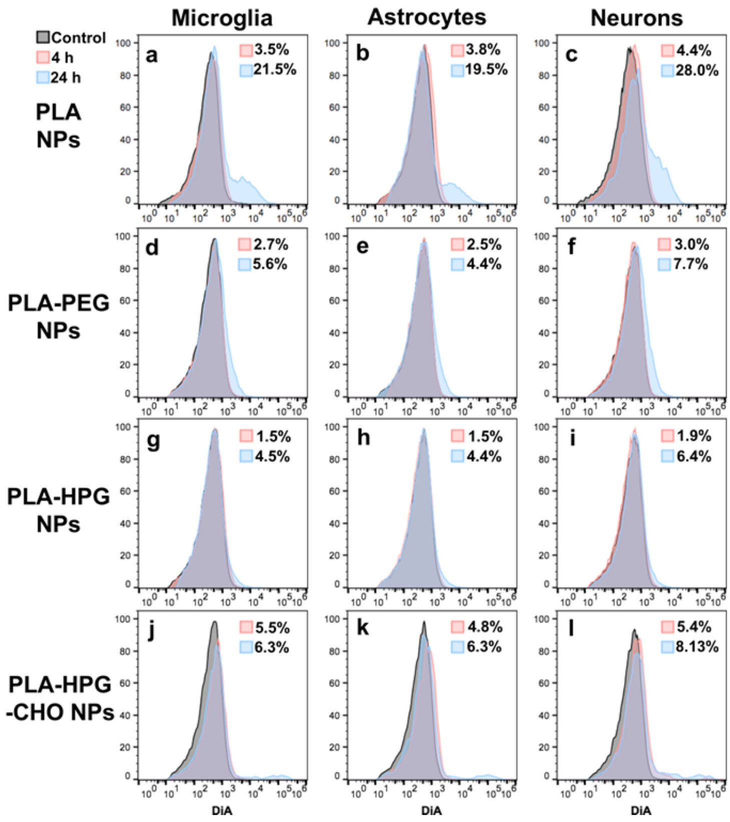

Supplementary Figure 4: Cell population shift in healthy brains. (a-l) Representative histograms of population shift of cells taking up NPs loaded with DiA at 4 h and 24 h (red and blue respectively). The percent shift is an average of N = 5 biological replicates (experiments of same particle type were done on different days to ensure reproducibility of processing, two control brains were harvested each day) and the MFI of the shifted population is displayed in **Fig 2a** and **Supplementary Fig 5**.

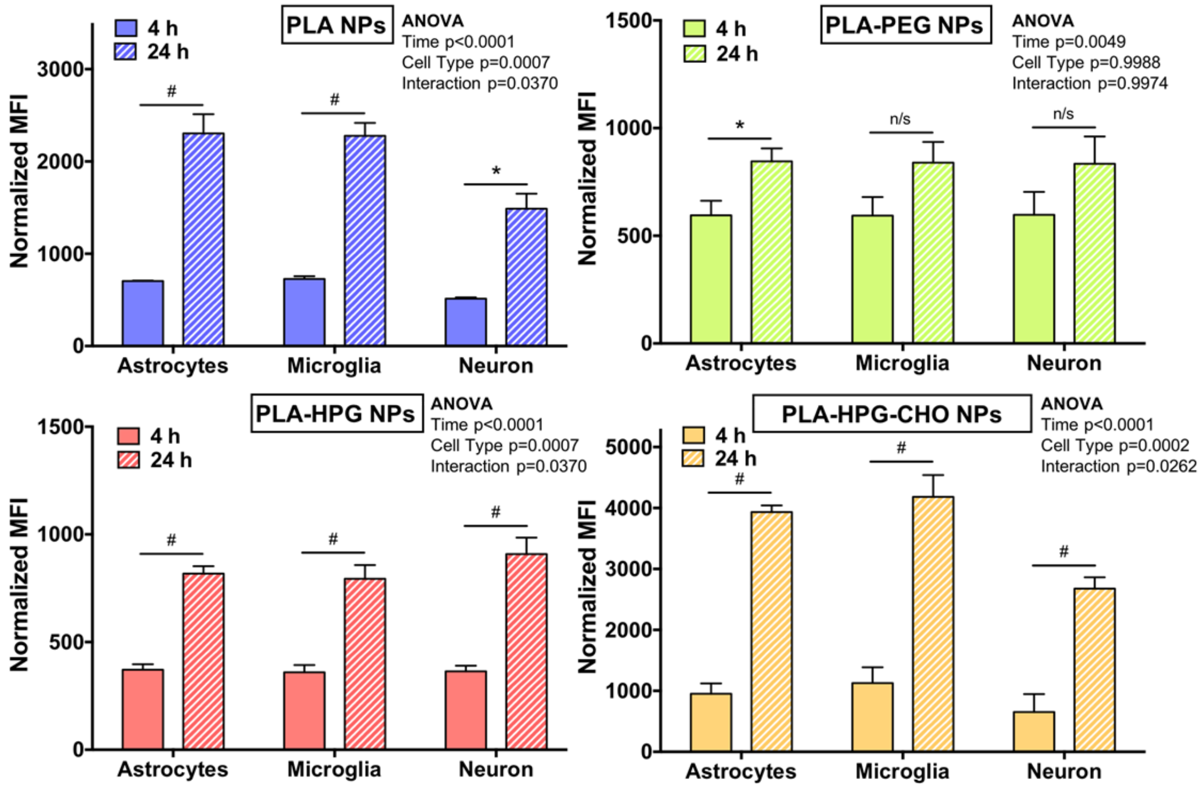

Supplementary Figure 5: Cellular tropism of NPs 4 h and 24 h after CED in the healthy brain. Mean fluorescence intensity of cell populations measured by flow cytometry 4 h and 24 h after CED treatment (results are presented as mean ± SD of N = 5 biological replicates, experiments of same particle type were done on different days to ensure reproducibility of processing, two control brains were harvested each day, statistical analysis was performed using a two sided student's t-test, *p < 0.05, #p < 0.005) and two-way ANOVA (displayed in figure and in detail in supplementary document).

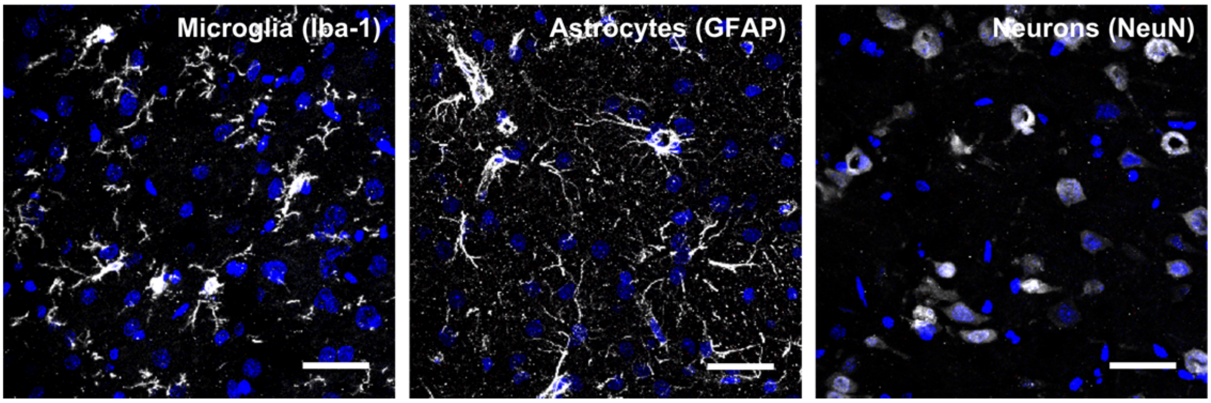

Supplementary Figure 6: Representative images of untreated brains stained for different cell populations. A healthy brain was stained for microglia (Iba-1), astrocytes (GFAP) and neurons (NeuN) to show baseline cellular morphology and density. All images were stained with DAPI (blue) for nuclear visualization. (Scale bar = 50μm).

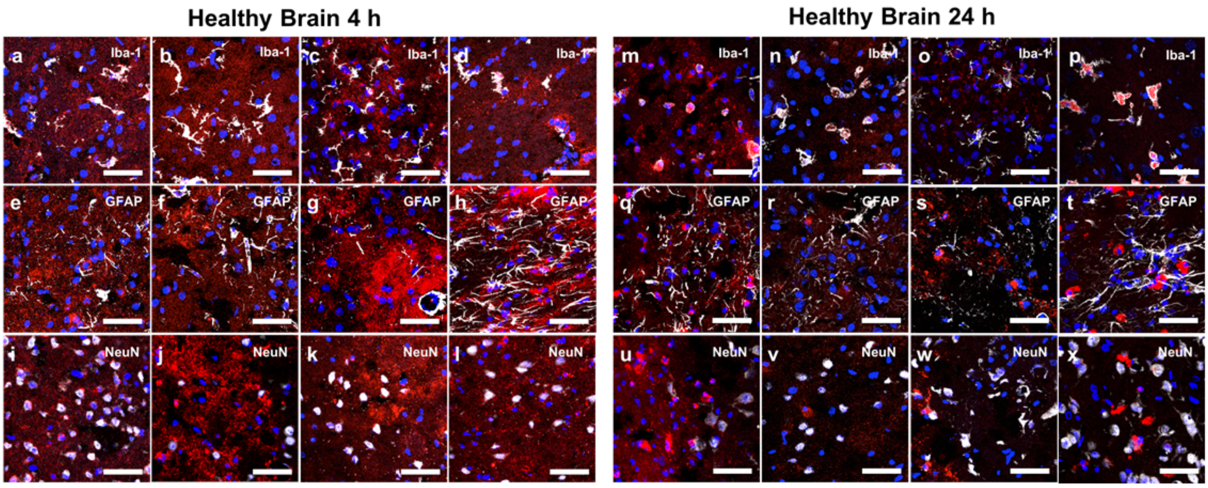

Supplementary Figure 7: Confocal images of healthy brain 4 h and 24 h after CED. Representative images taken with confocal microscopy of a healthy brain injected with PLA (**a,e,i,m,q,u**), PLA-PEG (**b,f,j,n,r,v**), PLA-HPG (**c,g,k,o,s,w**) and PLA-HPG-CHO (d,h,l,p,t,x) NPs (red). Tissue slices were stained using immunohistochemistry to look at change in morphology and/or uptake of particles by microglia, astrocytes and neurons (Iba-1, GFAP, and NeuN respectively, white). Ipsilateral and contralateral hemisphere images were taken to make sure of consistent staining between slices. Images confirmed that all particles types were internalized by all cell types, and different surface coatings only significantly modified microglia morphology as described in **Fig 2**. For each particle type and cell type the image is representative of three slides from one animal. All images were stained with DAPI (blue) for nuclear visualization. (Scale bar = 50μm).

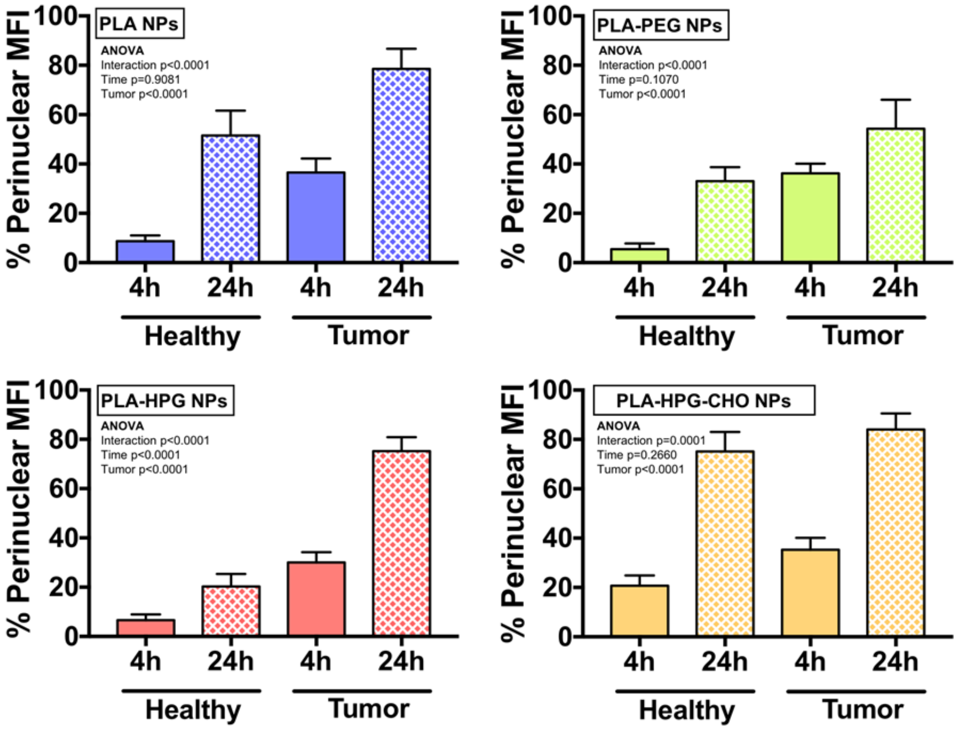

Supplementary Figure 8: Quantification of perinuclear NPs. Confocal images were analyzed using ImageJ. Cell nuclei were located in the DAPI channel, and circles with a diameter of 15 μm were drawn around each nuclei. Circles with MFI values higher than background in the DiA channel were selected as 'cells with perinuclear uptake', and the MFI in those circles was extracted. The % perinuclear MFI was calculated as the ratio of the MFI of 'cells with perinuclear uptake' over the total MFI of the image. Statistical analysis was performed using a two-way ANOVA (displayed in figure and in detail in supplementary document).

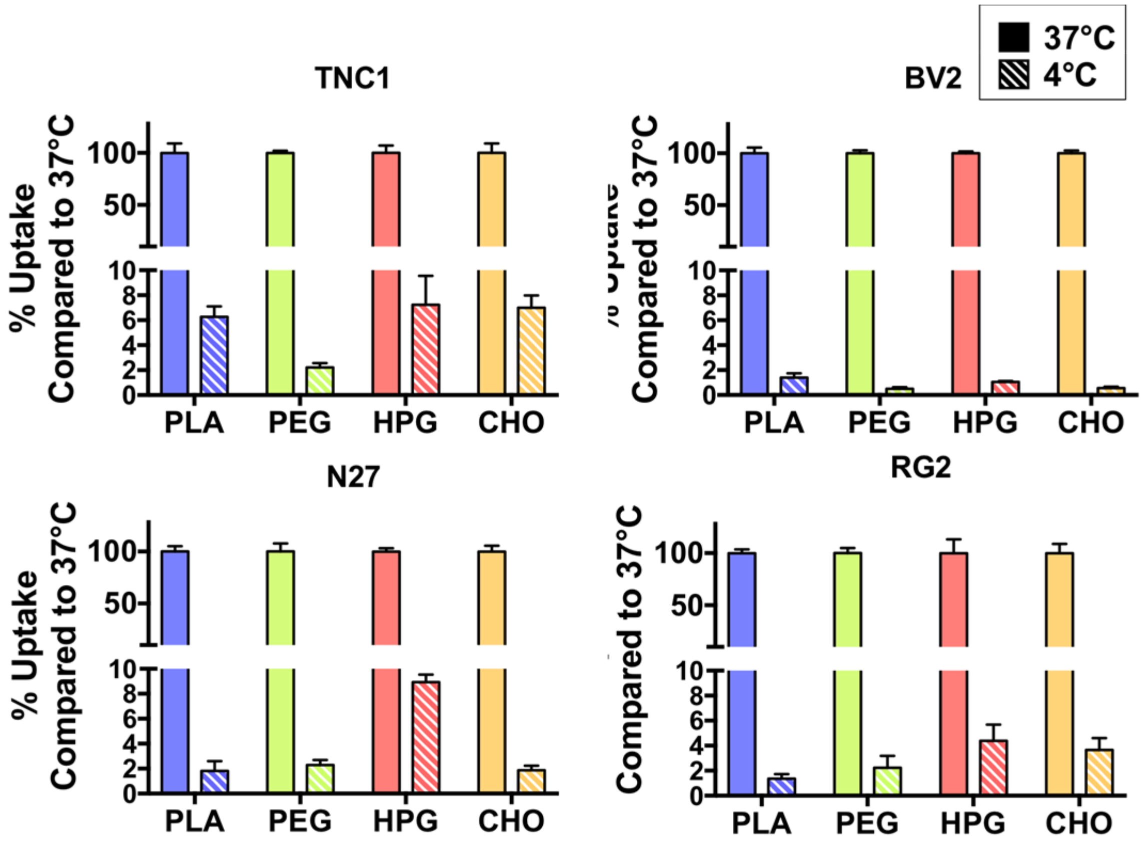

Supplementary Figure 9: In vitro cellular uptake at 37°C and 4°C. The four formulations were incubated with representative cell lines of the different cell types found in the brain (TNC-1 cells for astrocytes, BV-2 cells for microglia and N27 cells for neurons), for 4 h at 37°C (plain bars) or 4°C (striped bars). Cellular internalization was assessed by flow cytometry. For all cell types and all formulations, the uptake at 4°C did not exceed 10% of the uptake at 37°C, confirming an active internalization of all NPs formulation by the brain cells.

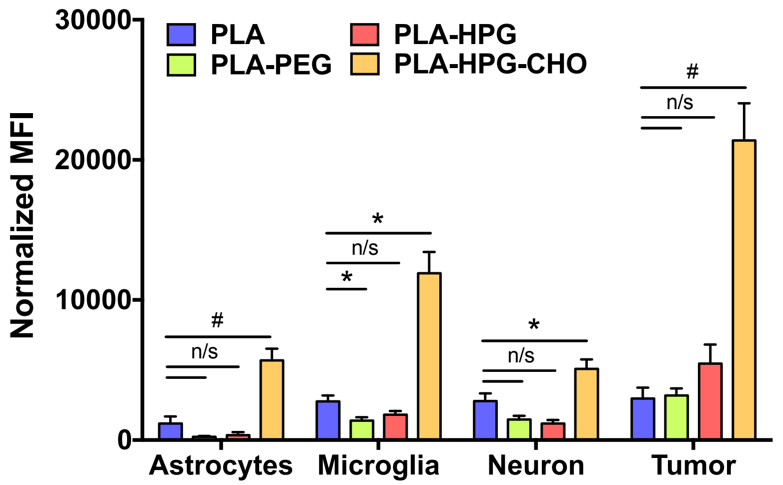

Supplementary Figure 10: Cellular tropism of NPs 4 h after CED in the tumor bearing brain. Normalized mean fluorescence intensity of cell populations measured by flow cytometry 4 h after CED treatment (results are presented as mean ± SD of N = 5 biological replicates, experiments of same particle type were done on different days to ensure reproducibility of processing, two control brains were harvested each day, statistical analysis was performed using a two sided student's t-test, *p < 0.05, #p < 0.005).

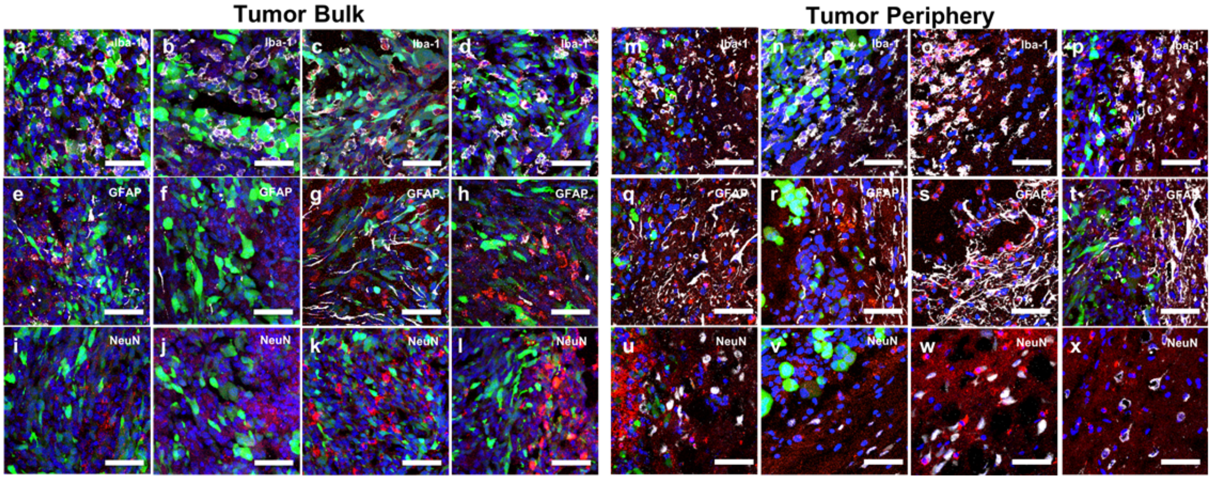

Supplementary Figure 11: Confocal images of tumor-bearing brain brain 4 h after CED. Representative images taken with confocal microscopy of a tumor-bearing (green) brain injected with PLA (**a,e,i,m,q,u**), PLA-PEG (**b,f,j,n,r,v**), PLA-HPG (**c,g,k,o,s,w**) and PLA-HPG-CHO (d,h,l,p,t,x) NPs (red). Tissue slices were stained using immunohistochemistry to look at change in morphology and/or uptake of particles by microglia, astrocytes and neurons (Iba-1, GFAP, and NeuN respectively in white). Representative images were taken in the tumor bulk (a) and at the tumor periphery (b) Ipsilateral and contralateral hemisphere images were taken to make sure of consistent staining between slices. For each particle type, cell type and area, the image is representative of three slides from one animal. (Scale bar = 50μm).

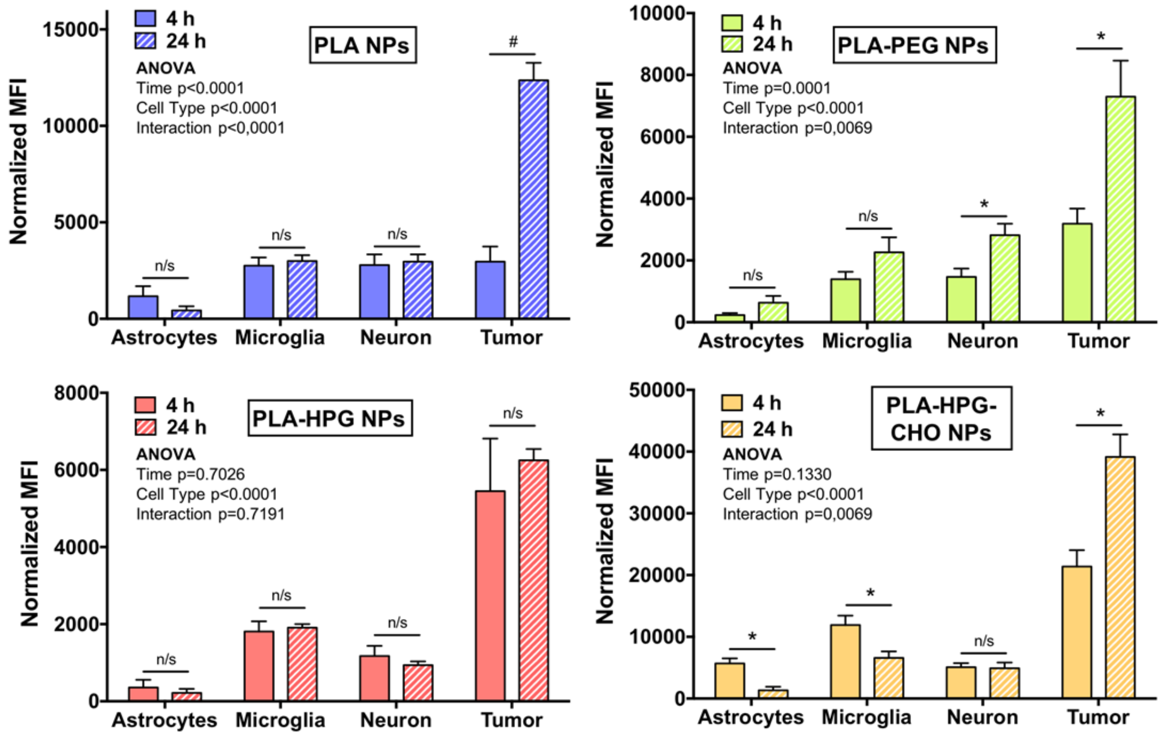

Supplementary Figure 12: Cellular tropism of NPs 4 h and 24 h after CED in the tumor-bearing brain. Rats were injected via CED 7 d after implantation of 250,000 RG2-GFP cells. Mean fluorescence intensity of each cell population in the DiA channel was measured with flow cytometry (results are presented as mean ± SD of N = 5 biological replicates, experiments of same particle type were done on different days to ensure reproducibility of processing, two control brains were harvested each day, statistical analysis was performed using a two sided student's t-test, $*p < 0.05$, $\#p < 0.005$) and two-way ANOVA (displayed in figure and in detail in supplementary document).

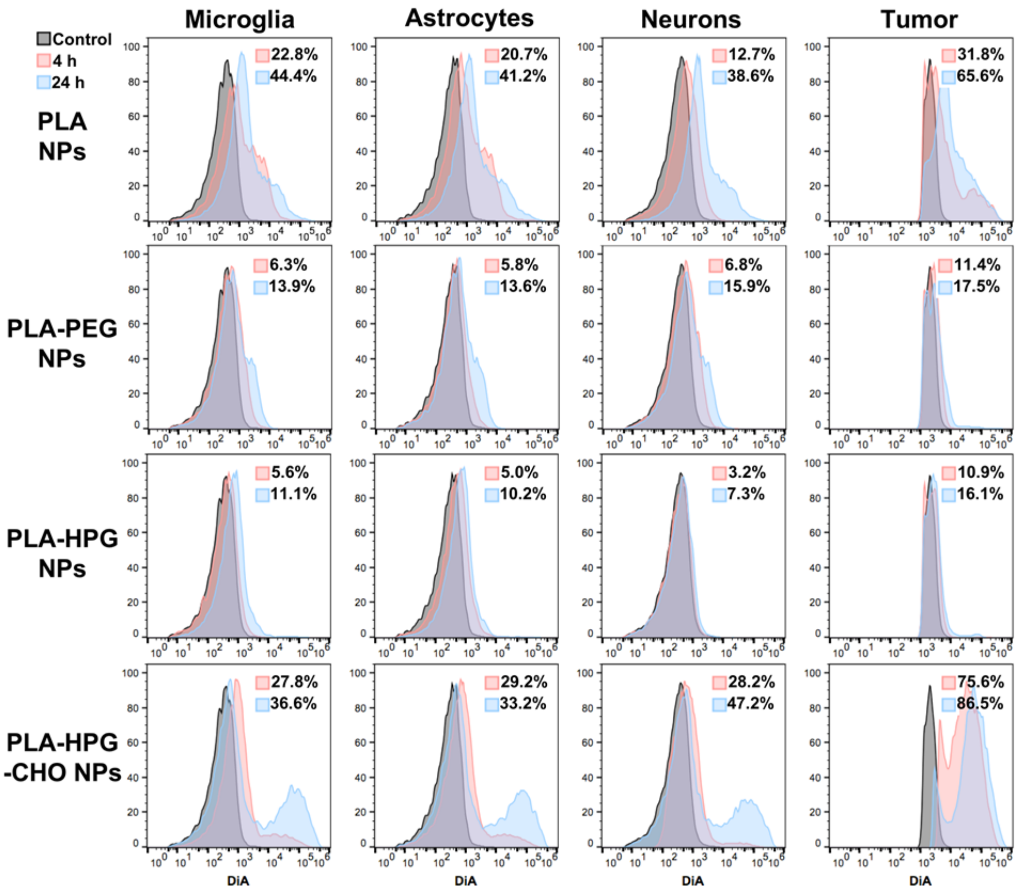

Supplementary Figure 13: Cell population shift in tumor bearing brains. Representative histograms of population shift of cells taking up NPs loaded with DiA at 4 h and 24 h (red and blue respectively). The percent shift is an average of N = 5 biological replicates (experiments of same particle type were done on different days to ensure reproducibility of processing, two control brains were harvested each day).

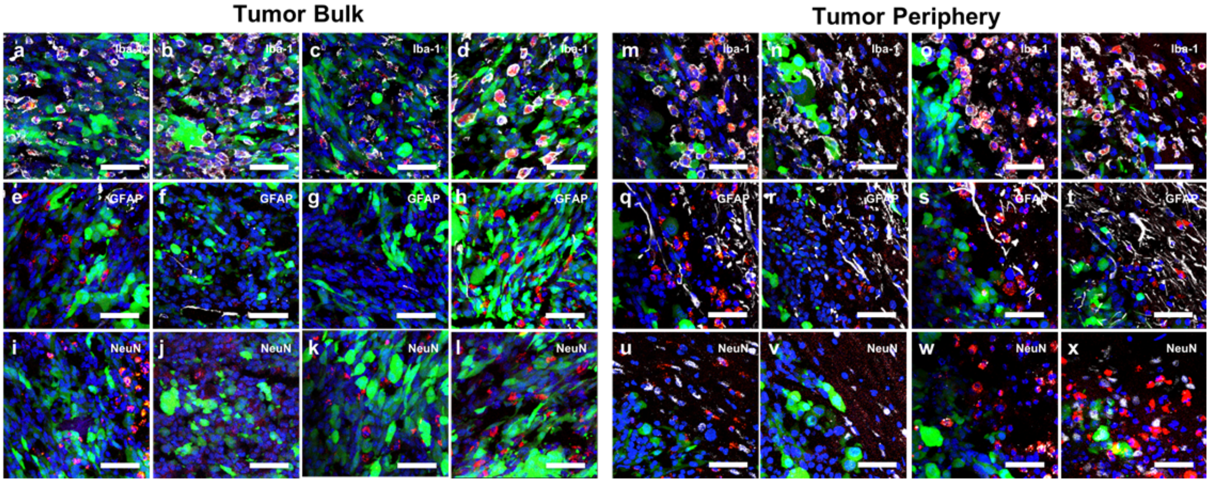

Supplementary Figure 14: Confocal images of tumor-bearing brain brain 24 h after CED. Representative images taken with confocal microscopy of a tumor-bearing (green) brain injected with PLA (**a,e,i,m,q,u**), PLA-PEG (**b,f,j,n,r,v**), PLA-HPG (**c,g,k,o,s,w**) and PLA-HPG-CHO (d,h,l,p,t,x) NPs (red). Tissue slices were stained using immunohistochemistry to look at change in morphology and/or uptake of particles by microglia, astrocytes and neurons (Iba-1, GFAP, and NeuN respectively in white). Representative images were taken in the tumor bulk (a) and at the tumor periphery (b) Ipsilateral and contralateral hemisphere images were taken to make sure of consistent staining between slices. For each particle type, cell type and area, the image is representative of three slides from one animal. (Scale bar = 50μm).

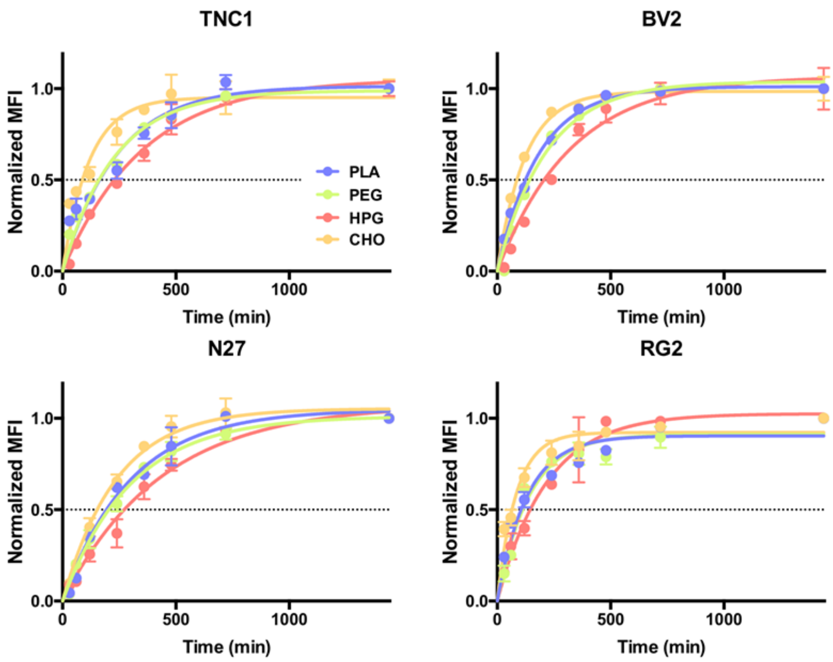

Uptake rates	PLA NPs	PLA-PEG NPs	PLA-HPG NPs	PLA-HPG-CHO NPs
**TNC1**	1.82 ± 0.22	1.91 ± 0.12	1.23 ± 0.08	3.71 ± 0.43
**BV2**	2.40 ± 0.09	2.00 ± 0.15	1.33 ± 0.13	3.56 ± 0.15
**N27**	1.46 ± 0.11	1.45 ± 0.07	1.00 ± 0.08	1.84 ± 0.11
**RG2**	3.33 ± 0.34	3.09 ± 0.31	2.00 ± 0.18	5.22 ± 0.50

R^2 values	PLA NPs	PLA-PEG NPs	PLA-HPG NPs	PLA-HPG-CHO NPs
**TNC1**	0.9024	0.9745	0.9801	0.8746
**BV2**	0.9921	0.9758	0.9609	0.9870
**N27**	0.9759	0.9875	0.9739	0.9816
**RG2**	0.9111	0.9400	0.9536	0.8927

Supplementary Figure 15: Uptake kinetics of NPs in different cell lines. Brain cells of rodent origin (TNC1 astrocyte, BV2 microglia, N27 neuronal, RG2 tumor) were treated with NPs for different time points and MFI was measured using flow cytometry. Association kinetics equation in Prism 6 was fitted to measure a normalized uptake rate **(Fig 4b)** (N = 3, biological replicates, experiment was repeated twice for reproducibility, s.d.).

Supplementary Table 1: Internalization of NPs 4 h and 24 h after CED in the healthy brain and the tumor-bearing brain. For each condition, mean fluorescence intensities of each cell population are reported. Each MFI has been normalized to the total internalization of naked PLA NPs in the healthy brain, 4 h after CED, in order to easily compare internalization levels between particle types, CED conditions (healthy brain vs tumor-bearing brain, 4 h vs 24 h), and cell type. PLA-HPG-CHO NPs displayed the highest internalization level in all conditions, while stealth particles (PLA-PEG and PLA-HPG NPs) presented similar low internalization.

	PLA NPs	PLA-PEG NPs	PLA-HPG NPs	PLA-HPG-CHO NPs	
**Healthy 4 h**	0.40	0.36	0.22	0.57	Astrocytes
	0.36	0.27	0.16	0.50	Microglia
	0.24	0.29	0.18	0.32	Neurons
	**1.00**	**0.92**	**0.56**	**1.40**	**Total**
**Healthy 24 h**	1.38	0.50	0.49	2.28	Astrocytes
	1.03	0.37	0.36	1.94	Microglia
	0.75	0.41	0.44	1.33	Neurons
	**3.13**	**1.29**	**1.29**	**5.55**	**Total**
**Tumor 4 h**	0.52	0.12	0.17	2.45	Astrocytes
	1.89	0.97	1.27	9.50	Microglia
	1.38	0.73	0.61	2.76	Neurons
	1.89	2.05	3.53	15.64	Tumor
	**5.74**	**3.86**	**5.52**	**30.66**	**Total**
**Tumor 24 h**	0.28	0.36	0.14	0.82	Astrocytes
	1.66	1.08	0.92	3.69	Microglia
	1.25	1.26	0.43	1.23	Neurons
	10.54	6.29	5.61	34.87	Tumor
	**13.87**	**8.98**	**7.10**	**41.02**	**Total**

Supplementary Table 2: Drug loading, characteristics and therapeutic efficacy of epothilone B (EB) loaded NPs. Particle characterization with dynamic light scattering and laser doppler anemometery displayed similar hydrodynamic diameters and zeta potential respectively for all particle types. Drug loading was measured using a LC-MS, and the mean survival time was measured with a survival study done on RG-2 tumor bearing rats.

	**PBS**	**Free EB**	**PLA NPs**	**PLA-PEG NPs**	**PLA-HPG NPs**	**PLA-HPG-CHO NPs**
**Hydrodynamic Diameter (nm)**	n/a	n/a	135 ± 1	137 ± 2	126 ± 1	128 ± 1
**PdI**	n/a	n/a	0.1	0.1	0.1	0.1
**ζ-Potential (mV)**	n/a	n/a	-25 ± 0.3	-20 ± 0.4	-15 ± 0.2	-22 ± 0.3
**Drug loading (%)**	n/a	n/a	2	2	2	2
**Mean survival time (days)**	16	18	33	21.5	28	28

Supplementary Table 3: Two-way ANOVA analysis of Supplementary Figure 5 and 12 comparing Particle and Cell type. Detailed ANOVA tables looking at statistical significance of mean differences between cell types and particle types. Graphs are provided in **Supplementary Figure 5 and 12** and additional analysis is shown in supporting documents.

Healthy 4 h	SS	DF	MS	F (DFn, DFd)	P value	P value summary	% of total variation
Interaction	323891	6	53982	F (6, 36) = 2.292	P=0.0563	ns	10.02
Cell Type	247684	2	123842	F (2, 36) = 5.259	P=0.0099	**	7.661
Particle Type	1813699	3	604566	F (3, 36) = 25.67	P<0.0001	****	56.1
Residual	847699	36	23547				

Healthy 24 h	SS	DF	MS	F (DFn, DFd)	P value	P value summary	% of total variation
Interaction	4020161	6	670027	F (6, 36) = 6.599	P<0.0001	****	5.576
Cell Type	2925258	2	1462629	F (2, 36) = 14.41	P<0.0001	****	4.057
Particle Type	61497714	3	20499238	F (3, 36) = 201.9	P<0.0001	****	85.3
Residual	3655171	36	101533				

Tumor 4 h	SS	DF	MS	F (DFn, DFd)	P value	P value summary	% of total variation
Interaction	382291117	9	42476791	F (9, 51) = 12.64	P<0.0001	****	19.67
Cell Type	389747973	3	129915991	F (3, 51) = 38.66	P<0.0001	****	20.05
Particle Type	978498652	3	326166217	F (3, 51) = 97.05	P<0.0001	****	50.34
Residual	171398905	51	3360763				

Tumor 24 h	SS	DF	MS	F (DFn, DFd)	P value	P value summary	% of total variation
Interaction	1823016690	9	202557410	F (9, 48) = 43.91	P<0.0001	****	32.62
Cell Type	2398369850	3	799456617	F (3, 48) = 173.3	P<0.0001	****	42.92
Particle Type	1145577083	3	381859028	F (3, 48) = 82.77	P<0.0001	****	20.5
Residual	221445405	48	4613446				
